# Upregulated immuno-modulator PD-L1 in malignant peripheral nerve sheath tumors provides a potential biomarker and a therapeutic target

**DOI:** 10.1007/s00262-020-02548-1

**Published:** 2020-03-19

**Authors:** Said Farschtschi, Lan Kluwe, Su-Jin Park, Su-Jun Oh, Nancy Mah, Victor-Felix Mautner, Andreas Kurtz

**Affiliations:** 1 Department of Neurology, University Medical Center Hamburg-Eppendorf, Martinistr. 52, 20246 Hamburg, Germany; 2 BIH Center for Regenerative Therapies, Charité University Medicine Berlin, Augustenburger Platz 1, 13353 Berlin, Germany

**Keywords:** Neurofibromatosis type 1, Biomarker, ELISA, Malignant peripheral nerve sheath tumor, PD-L1, Immuno-modulator

## Abstract

**Background:**

Malignant peripheral nerve sheath tumors (MPNSTs) are rare aggressive sarcomas with poor prognosis. More than half of MPNSTs develop from benign precursor tumors associated with neurofibromatosis type 1 (NF1) which is a tumor suppressor gene disorder. Early detection of malignant transformation in NF1 patients is pivotal to improving survival. The primary aim of this study was to evaluate the role of immuno-modulators as candidate biomarkers of malignant transformation in NF1 patients with plexiform neurofibromas as well as predictors of response to immunotherapeutic approaches.

**Methods:**

Sera from a total of 125 NF1 patients with quantified internal tumor load were included, and 25 of them had MPNSTs. A total of six immuno-modulatory factors (IGFBP-1, PD-L1, IFN-α, GM-CSF, PGE-2, and AXL) were measured in these sera using respective ELISA.

**Results:**

NF1 patients with MPNSTs had significantly elevated PD-L1 levels in their sera compared to NF1 patients without MPNSTs. By contrast, AXL concentrations were significantly lower in sera of NF1-MPNST patients. IGFBP-1 and PGE2 serum levels did not differ between the two patient groups. IFN-α and GM-CSF were below the detectable level in most samples.

**Conclusion:**

The immuno-modulator PD-L1 is upregulated in MPNST patients and therefore may provide as a potential biomarker of malignant transformation in patients with NF1 and as a response predictor for immunotherapeutic approaches.

**Electronic supplementary material:**

The online version of this article (10.1007/s00262-020-02548-1) contains supplementary material, which is available to authorized users.

## Introduction

Malignant peripheral nerve sheath tumors (MPNSTs) are aggressive sarcomas with very poor prognosis and limited therapeutic approaches. More than half of the MPNSTs develop from plexiform neurofibromas (PNF) in patients with neurofibromatosis type 1 (NF1), an autosomal-dominant neurocutaneous tumor suppressor gene disorder [1–3]. PNFs themselves are benign tumors and can be found in more than half of NF1 patients. However, PNFs have a high risk of malignant transformation into MPNSTs. Consequently, NF1 patients have a high lifetime risk (8–13%) of developing MPNSTs, which are the major cause for their reduced life span [1, 2, 4, 5].

Unfortunately, chemotherapy and radiotherapy can hardly improve survival of MPNST patients. So far, complete surgical resection is the solely reliable curative treatment for MPNSTs, which is only possible for cases with timely diagnosis [2, 6]. Noninvasive biomarkers for early detection of malignant transformation would therefore be valuable.

In a recent study, we found high serum concentrations of two immuno-modulators in NF1 patients with MPNSTs: the insulin-like growth factor-binding protein 1 (IGFBP-1) and the Chemokine Regulated upon Activation, Normal T cell Expressed and Secreted (RANTES) [7]. Moreover, we found an inverse correlation of CD8(+)/CD57(+) and CD27(−) T cell fractions with benign internal tumor load [8]. These findings raised the hypothesis of a potential systemic inflammatory response in the tumor microenvironment which may accompany tumor progression and contribute to malignant transformation in NF1 patients. In light of these studies, we analyzed an array of immuno-modulatory proteins, which have been associated with tumor phenotypes and could be exploited for tumor therapy, including IGFBP-1, programmed cell death ligand 1 (PD-L1), the soluble tyrosine receptor kinase AXL, interferon-α (IFN-α), granulocyte-macrophage colony-stimulating factor (GM-CSF), and prostaglandin E2 (PGE2).

Studies reported that binding of PD-L1 to its receptor programmed cell death protein 1 (PD-1) regulate an immune checkpoint, which then suppresses T cell cytotoxicity [9]. Serum PD-L1 is also reported as a biomarker for immune suppression in patients with other tumors [10, 11]. Increased PD-1 and PD-L1 expression would provide a rationale to test the efficacy of immune checkpoint inhibitors/immune therapies targeting this pathway [12, 13].

AXL is a receptor tyrosine kinase which regulates various vital cellular processes including proliferation, survival, motility, and immunologic response. AXL is overexpressed in several malignancies and was postulated to contribute to oncogenic processes including angiogenesis and therapy resistance to and suppression of immune response. Therefore, AXL is considered as a potential prognostic biomarker for malignancy and as a target for therapy development. AXL has emerged as a key facilitator of immune escape and drug-resistance by cancer cells, leading to aggressive and metastatic cancers [14, 15]. In a previous study, NF1 patients with PNFs showed increased soluble AXL levels in serum, compared to NF1 patients with only dermal neurofibroma [16].

IFN-α is a possible inhibitor of tumorigenesis as it stimulates the immune response against tumor cells [17], and thus we speculated that it may be downregulated in MPNST patients. GM-CSF plays a critical role in immune modulation and hematopoiesis; however, it is also frequently upregulated in multiple human cancer types, marking these recognizable for the immune system to stimulate dendritic cell (DC) maturation and monocyte/macrophage activity, but it also seems to stimulate tumor growth and metastasis [18]. PGE2 is a hormonal immune regulator, which is also known to suppress T cell receptor signaling [19].

In this background, we design the present study to measure six selected immuno-modulators and potential activators of MPNSTs—tumorigenesis in sera of a total of 125 NF1 patients, among them 25 had MPNSTs: PD-L1, IGFBP-1, AXL, IFN-α, GM-CSF, and PGE2.

## Materials and methods

### Sample collection

All the 125 patients were ascertained for the diagnosis of NF1 followed the NIH criteria [20], among them 25 had histologically confirmed MPNSTs. Serum samples from these patients were obtained in the Neurofibromatosis Outpatient Clinic of the Department of Neurology at the University Medical Center Hamburg-Eppendorf, Germany. Serum preparation followed strictly the standardized protocol: Venous blood of up to 20 ml was collected and separated by centrifugation exactly 30 min later. Serum was then immediately frozen in 100 µl aliquots and stored at − 80 °C until use.

## Determination of tumor load

Whole-body MRI was performed in NF1-patients to quantify total internal tumor volume. MRI and calculation of total tumor volume were performed in analogy to previous studies [8, 21, 22]. All patients underwent an identical scan protocol with the same parameters for slice thickness, gap, orientation, field of view, imaging matrix, image resolution, echo time, repetition time, and inversion time (Siemens Avanto 1.5 T). The subjects were imaged in a supine position from head to knee in four steps (head, thorax, abdomen, and legs) in accordance with the maximum range of table movement. Slice thickness is 5–10 mm without skips between slices. MRI analysis was performed by an experienced radiologist and a physician trained in image analysis of NF1-associated tumors (Said Farschtschi). The analysis was carried out in a blinded manner. Tumor segmentation and volumetry were performed semiautomatically with the heuristics-based software MEDx (V 3.44) using fat-suppressed T2-short tau inversion recovery sequences (T2-STIR). Differences in signal intensity of neurofibroma tissue and surrounding tissue were used to define tumor margins on axial slices. The method used for this automated volumetric analysis is sensitive (it detects volume changes as small as 10%), reproducible (coefficient of variation 0.6%–5.6%), and produces results similar to manual tumor tracings (*R* = 0.999). Patients were divided in the groups high (> 500 ml), medium (50–300 ml) and low/no (< 10 cm^3^) internal tumor.

## Enzyme-linked immunosorbent assays (ELISA)

ELISA were carried out using the protocol of the manufacturer. The used kits were for AXL1, GM-CSF, IFN-α, IGFBP-1 (all RayBiotech, ELH, USA), PGE2 (Abnova KA0326, Japan), and PD-L1 (Affymetrix/Thermo Fischer BM2212). Serum concentration of each of these six immuno-modulators was compared between NF1 patients with and without MPNSTs using a *t* test with two-tailed hypothesis and equal variation.

### Statistical analysis

Statistical analysis was carried out using unpaired *t* test with a two-tailed hypothesis. Significant level was set at 0.05.

## Results

**Fig. 1:**
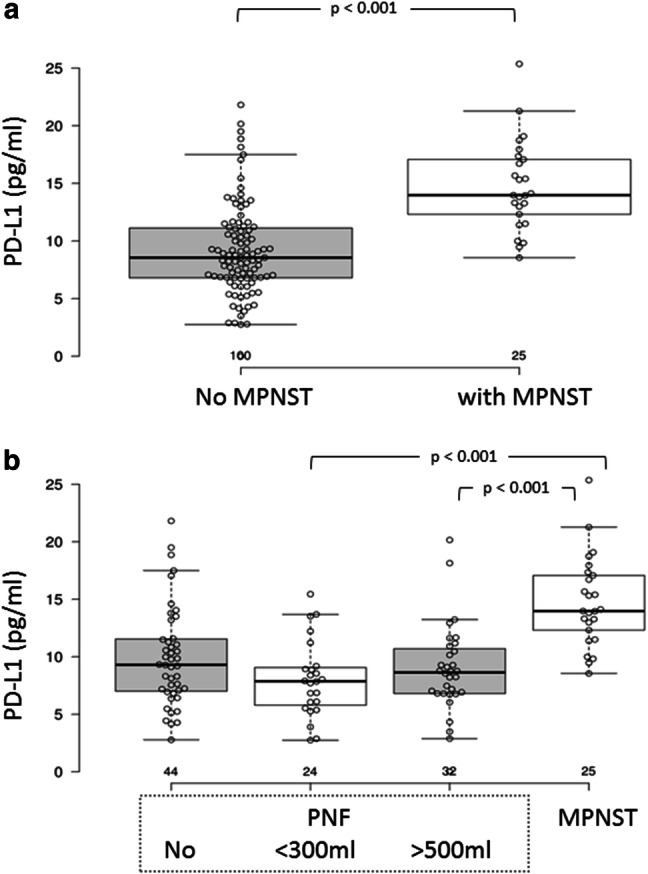
Serum PD-L1 concentration in NF1 patients with and without MPNSTs. **a** Box plot; **b** distribution in patients with no MPNSTs;**c** distribution in patients with MPNSTs

PD-L1 was significantly elevated in sera of NF1 patients with MPNSTs compared to NF1 patients without MPNSTs (Fig. 1a). In NF1, the presence of a PNF is a prerequisite to develop an MPNSTs [1]. When we stratified the patient cohorts without MPNSTs but with PNF into groups of > 500 ml and < 300 ml internal PNF tumor load, and those with no internal tumors, additional patterns emerged. PD-L1 was only increased in serum of MPNST patients when compared to patients with PNF, while the PD-L1 levels in NF1 patients without internal tumors was highly variable (Fig. 1b). The distribution is nearly normal.

**Fig. 2:**
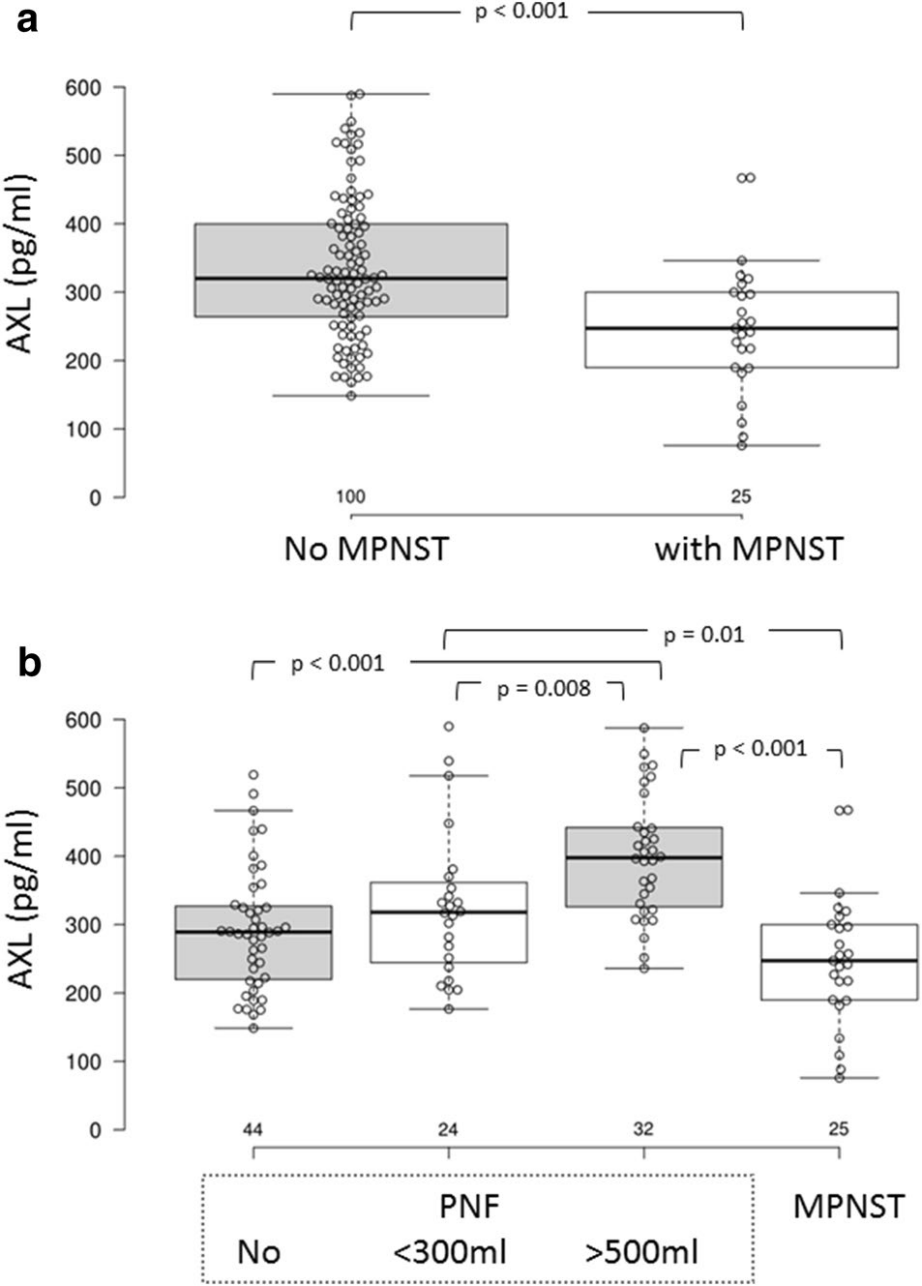
Serum AXL concentration in NF1 patients with and without MPNSTs. **a** Box plot; **b** distribution in patients with no MPNSTs; C: distribution in patients with MPNSTs

By contrast, AXL serum concentrations were significantly reduced in MPNST patients compared to patients without MPNSTs (Fig. 2a). When patients without MPNSTs were divided into three groups according to their internal PNF tumor loads, the two groups with PNF loads under 300 ml had significantly lower AXL concentration than patients with high tumor load (> 500 ml). By contrast, AXL concentration did not differ significantly between patient without PNF and patient with MPNSTs (Fig. 2b).

**Fig. 3:**
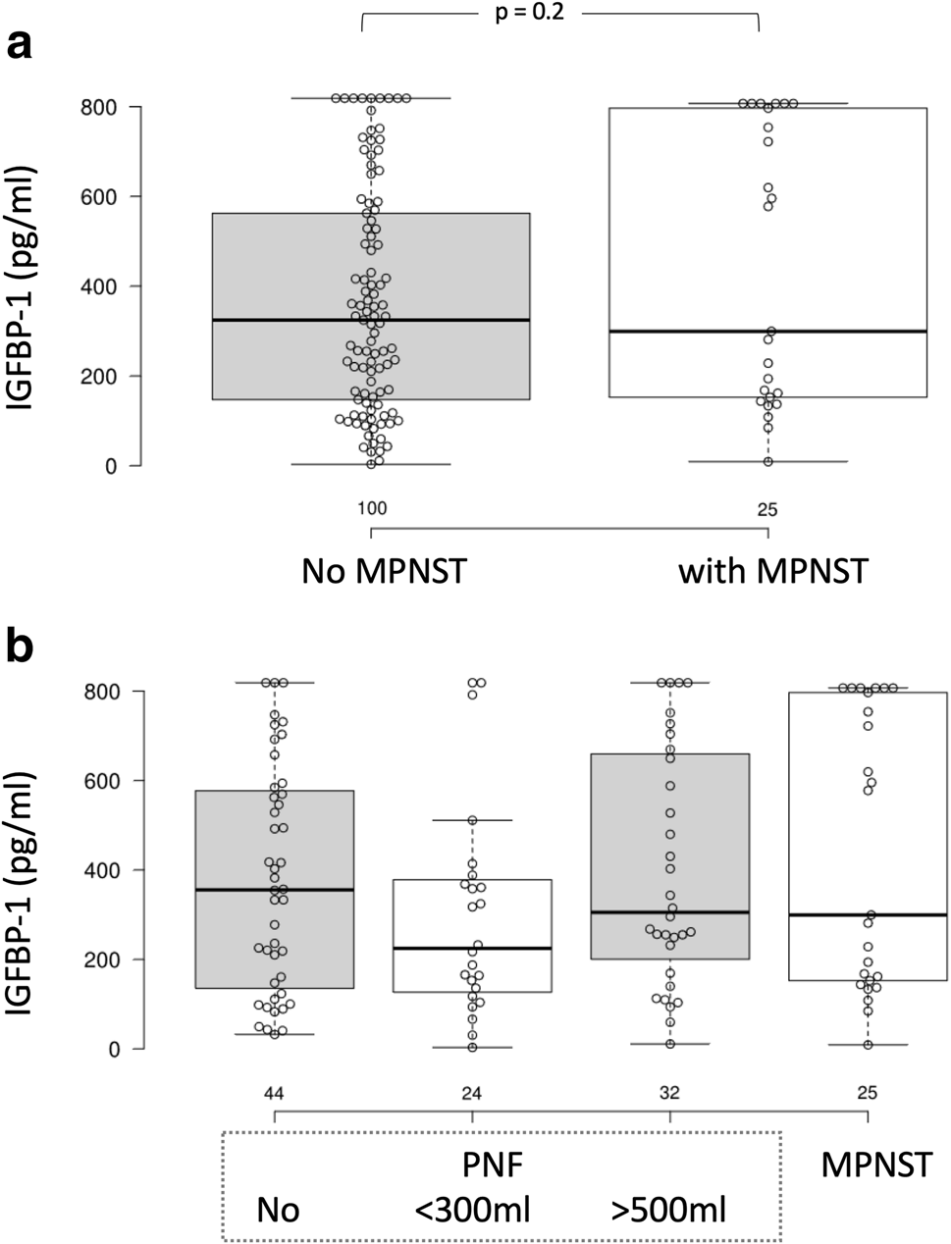
Serum IGFBP-1 concentration in NF1 patients with and without MPNSTs. **a** Box plot; **b** distribution in patients with no MPNSTs; C: distribution in patients with MPNSTs

**Fig. 4:**
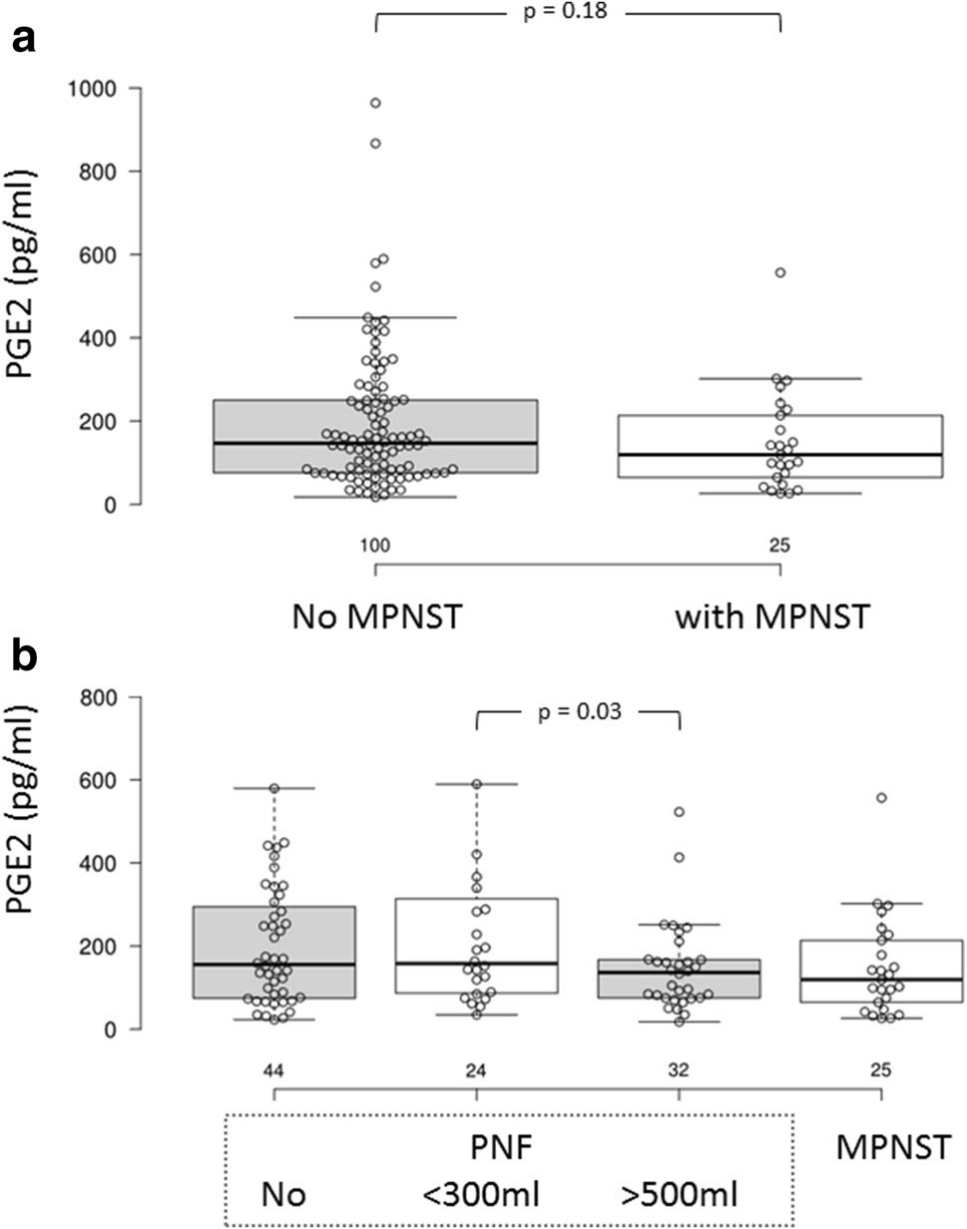
Serum PGE2 concentration in NF1 patients with and without MPNSTs. **a** Box plot; **b** distribution in patients with no MPNSTs; C: distribution in patients with MPNSTs

IGFBP-1 and PGE2 serum concentrations did not differ between the patients with and without MPNSTs (Figs. 3a, 4a). Interestingly, MPNST patients are clearly divided into two groups regarding IGFBP-1 serum concentration: (1) 50–300 pg/ml and (2) 600–850 pg/ml (Figs. 3b, 4b).

The other tested immuno-modulators IFN-α and GM-CSF were below the detectable level in most of the samples and meaningful analysis therefore not possible.

None of the immuno-modulators correlated with patient age. AXL was slightly higher in male patients (*P* = 0.03). All other modulators did not differ between male and female patients (data not shown).

## Discussion

The major and most important finding of the present study is the highly significantly elevated serum level of the immuno-modulator PD-L1 in NF1 patients with MPNSTs. Moreover, the PD-L1 levels are particularly increased compared to patients with PNF, which are of increased risk to develop MPNSTs. Thus, PD-L1 may be a biomarker for the development of an MPNSTs in this risk group, although we do currently not know whether early stages of malignant transformation can be detected, which would require longitudinal studies. The Programmed death 1 (PD-1) inhibitory receptor is expressed by effector cytotoxic T cells during long-term antigen exposure. Binding to its ligand, PD-L1 results in negative regulation of T cells. PD-L1 is primarily expressed in inflamed tissues and tumors. Thus, PD-L1 expression in the tumor microenvironment will reduce the T cell-based antitumor immune response [10, 23, 24]. Two anti-PD-1 monoclonal antibodies (nivolumab and pembrolizumab) [25–27] and three anti-PD-L1 monoclonal antibodies (atezolizumab, avelumab, and durvalumab) [28–30] have been approved by the US Food and Drug Administration (FDA) for treating cancers, for example, melanoma, non-small cell lung cancer, and renal cell carcinoma. Last year (after we completed the present study), PD-1 inhibition was reported to have achieved a complete metabolic response for a MPNSTs [31]. In addition, a trial with nivolumab and ipilimumab for rare tumors is also in progress [ClinicalTrials.gov NCT02834013]. Our finding of elevated serum levels of PD-L1 is in concordance with rational of such trials.

PD-L1 expression has been shown in circulating tumor cells, tumors and in monocytes, among others [32–39]. A recent study reported positive staining for PD-L1 in small portions (> 5%) of cells of a small portion (13%) of MPNSTs and that PD-L1 expression is less profound in benign nerve tumors including neurofibromas and schwannomas [40]. However, a more recent study did not find significant variation of the average PD-L1 staining between the MPNSTs and the benign neurofibromas [41]. In any case, we currently do not know which cells in NF1 patients with MPNSTs express and secrete PD-L1, leading to the increased serum concentration. Nevertheless, enhanced serum concentrations of PD-L1 have been indicative for attempting anti-PD-L1 is additional evidence supporting therapy [42].

We previously reported significant higher IGFBP-1 in serum of NF1 patients with MPNSTs than in NF1 patients without MPNSTs [7]. However, this finding was not confirmed in the present study including 25 MPNST patients, of which 15 showed increased IGFBP-1 serum levels (> 30 ng/ml). However, the MPNST cohort could be divided in two subgroups, one of which showed high IGFBP-1 serum levels. The phenotypic nature distinguishing these subgroups could not be identified, but is not due to variable PNF tumor burden. Our previous data of a reduced level of terminally differentiated CD8(+)/CD57(+) and CD27(−) T cell fractions in NF1 patients with high benign internal tumor load may indicate a reduced immune capacity already at the benign state, which is further exacerbated by the high PD L1 levels at the malignant state.

An unexpected finding was the significantly lowered serum level of another immuno-modulator AXL. Previous data indicated an increase in soluble AXL in patients with PNF. Our data confirmed this, and in addition, we could show that AXL serum levels are associated with internal tumor load.

Although the present study is providing strong evidence of a role for PD L1 in MPNST progression, it does not provide longitudinal and follow-up information. Future studies performed in a systematic manner to obtain samples before and after MPNST surgery, at multiple time points and longitudinal clinical assessment accompanied by immunohistochemical studies of the tumor tissue are needed to define the specific role of PD L1 for MPNST pathology. Since MPNSTs are rare tumors, multiple clinics and centers need to collaborate to enable reasonable number of cases, data and specimens.

In conclusion, the immuno-modulator PD-L1 is upregulated in MPNST patients which may provide a biomarker for early detection of malignant transformation in NF1 patients and may be a molecular target for developing new therapies.

### Electronic supplementary material

Below is the link to the electronic supplementary material.

Supplementary file1 (PDF 46 kb)

## Abbreviations

AXL: Soluble tyrosine receptor kinase AXL
FDA: Food and Drug Administration of the United States of America
IGFBP-1: Insulin-like growth factor-binding protein 1
MPNSTs: Malignant peripheral nerve sheath tumors
NF1: Neurofibromatosis type 1
PGE-2: Prostaglandin E2
RANTES: Chemokine Regulated upon Activation, Normal T cell Expressed and Secreted, also Chemokine ligand 5 (CCL5)
T2-STIR: T2-Short Tau Inversion Recovery
