# Supplementary material for: Upregulated immuno-modulator PD-L1 in malignant peripheral nerve sheath tumors provides a potential biomarker and a therapeutic target
Source: Cancer Immunol Immunother. 2020 Mar 19;69(7):1307–13. doi: 10.1007/s00262-020-02548-1 (PMC7303069; doi:10.1007/s00262-020-02548-1)
Supplement: Supplementary file 1 — Supplementary file1 (PDF 46 kb) [file 262_2020_2548_MOESM1_ESM.pdf]

Patient	Gender	Age	Axl	IGFBP-1	PD-L1	PGE2	Tumor load
1	male	36	406,2	295,6	8,8	161,6	high
2	male	34	516,3	726,8	10,2	63,9	high
3	male	32	304,9	169,1	11,2	92,4	high
4	male	23	532,9	231,6	10,9	167,0	high
5	female	39	363,3	818,6	8,2	34,7	high
6	male	20	393,7	261,4	12,9	139,6	high
7	male	48	392,7	479,6	13,2	249,7	high
8	female	22	549,4	818,6	18,1	81,9	high
9	male	24	587,5	402,6	4,3	133,0	high
10	male	70	425,1	704,0	20,2	105,1	high
11	male	19	530,1	255,0	8,7	149,9	high
12	female	32	367,8	588,1	2,9	233,8	high
13	male	23	415,3	59,3	6,8	75,5	high
14	female	32	396,4	343,2	7,0	413,2	high
15	female	41	508,6	267,6	6,9	74,9	high
16	male	45	319,1	255,1	7,2	155,1	high
17	female	23	440,6	103,2	6,0	96,3	high
18	female	19	408,6	314,5	8,5	522,6	high
19	male	29	306,1	10,7	6,8	73,3	high
20	female	21	235,9	109,5	3,5	83,8	high
21	male	20	442,9	649,6	10,5	162,0	high
22	male	24	330,4	751,3	8,2	167,7	high
23	female	50	399,1	818,6	11,7	47,1	high
24	male	17	321,6	249,2	11,6	50,4	high
25	female	51	280,1	139,7	6,8	17,6	high
26	female	22	251,5	669,5	9,3	142,1	high
27	male	20	492,3	527,3	0,0	251,3	high
28	male	47	354,2	430,3	9,3	84,5	high
29	male	21	434,4	93,7	9,1	211,1	high
30	male	17	307,3	112,5	8,8	69,9	high
31	male	27	344,8	818,6	6,7	244,4	high
32	male	22	421,7	256,1	7,5	160,3	high
33	male	33	369,7	360,7	7,8	34,6	medium
34	male	31	251,2	165,6	6,8	72,3	medium
35	female	33	332,2	232,3	13,5	420,6	medium
36	female	31	313,2	413,9	6,1	288,4	medium
37	male	64	268,3	357,9	8,9	143,6	medium
38	male	18	204,4	2,9	5,5	84,5	medium
39	male	31	327,0	30,4	8,4	866,7	medium
40	female	59	381,0	117,4	8,5	54,4	medium
41	female	33	301,9	152,9	5,4	163,3	medium
42	female	54	517,6	388,2	12,2	190,5	medium
43	male	27	319,1	510,9	2,7	282,8	medium
44	male	19	539,2	818,6	11,2	126,7	medium
45	male	23	353,3	368,1	8,9	963,9	medium
46	male	33	237,6	135,6	7,9	152,5	medium
47	male	42	589,8	187,4	8,0	118,7	medium

48	male	41	281,6	217,0	3,9	589,4	medium
49	female	25	176,5	818,6	9,2	366,2	medium
50	male	44	317,0	163,9	13,7	196,4	medium
51	male	35	447,8	66,2	5,3	61,3	medium
52	female	48	217,9	791,6	6,0	142,2	medium
53	female	40	204,5	317,3	2,9	228,1	medium
54	male	52	341,2	103,7	15,4	89,1	medium
55	female	21	331,9	93,6	6,9	339,6	medium
56	female	40	210,4	324,3	7,7	74,9	medium
57	male	34	439,5	42,8	11,1	270,7	no
58	female	30	286,3	89,0	10,3	248,1	no
59	male	37	290,3	277,4	10,5	135,3	no
60	male	56	203,4	494,0	7,5	441,6	no
61	female	58	249,6	417,6	9,3	283,9	no
62	female	39	189,5	82,4	4,4	131,8	no
63	female	44	354,5	356,7	13,8	448,5	no
64	female	31	222,3	123,2	5,1	349,1	no
65	male	40	382,0	110,8	8,3	41,1	no
66	male	33	466,5	724,8	2,8	343,1	no
67	male	25	519,1	584,6	11,3	169,3	no
68	male	28	282,5	818,6	6,4	253,6	no
69	female	61	491,0	99,9	7,2	88,6	no
70	female	48	189,1	818,6	7,2	73,2	no
71	female	51	265,5	49,4	9,8	305,7	no
72	male	58	359,3	40,6	6,9	22,7	no
73	female	39	324,9	569,5	4,3	323,0	no
74	female	40	217,5	92,4	5,2	579,3	no
75	male	44	294,8	31,9	5,5	169,2	no
76	male	50	262,5	594,0	6,4	67,1	no
77	female	36	307,1	703,0	11,6	345,2	no
78	female	66	176,9	218,6	4,1	66,0	no
79	female	25	167,9	562,4	9,3	437,0	no
80	female	45	320,8	657,4	6,8	26,9	no
81	female	35	290,5	235,7	9,2	122,6	no
82	female	42	316,8	818,6	9,8	389,1	no
83	male	25	285,6	382,2	8,1	236,6	no
84	female	55	277,3	731,3	7,1	158,8	no
85	female	24	295,8	160,6	10,0	220,8	no
86	female	52	324,7	692,0	7,6	141,3	no
87	female	55	437,1	492,1	10,8	60,5	no
88	female	26	386,8	545,9	9,1	416,2	no
89	female	26	235,6	225,6	17,0	152,4	no
90	female	41	244,2	402,5	13,2	248,2	no
91	female	50	214,0	332,9	21,8	65,0	no
92	female	41	175,1	747,0	14,0	141,1	no
93	male	48	328,8	220,6	11,5	67,7	no
94	male	27	400,4	210,1	18,9	174,3	no
95	female	60	289,5	333,1	14,6	83,6	no

96	female	53	296,4	528,6	19,5	98,9	no
97	female	47	148,4	98,1	10,6	76,2	no
98	female	46	175,8	416,2	13,5	34,7	no
99	female	40	288,4	354,3	8,3	31,7	no
100	female	57	195,5	147,1	17,5	114,8	no
101	male	47	346,1	577,6	19,1	149,1	mpnst
102	male	29	257,1	619,4	18,7	94,9	mpnst
103	male	24	216,9	136,9	15,7	227,5	mpnst
104	female	47	88,2	753,7	9,5	26,4	mpnst
105	female	47	467,4	806,9	21,3	119,3	mpnst
106	female	22	294,3	299,3	17,1	47,5	mpnst
107	male	31	189,8	152,4	17,3	213,5	mpnst
108	male	38	238,2	721,9	12,3	95,3	mpnst
109	male	47	311,4	595,5	13,3	178,4	mpnst
110	female	41	188,9	161,5	13,3	297,6	mpnst
111	female	55	133,7	806,9	8,5	142,0	mpnst
112	female	50	217,5	8,7	9,8	556,6	mpnst
113	female	55	299,9	193,8	13,9	64,8	mpnst
114	female	25	324,5	167,7	11,5	301,9	mpnst
115	male	45	255,3	806,9	17,9	242,7	mpnst
116	male	43	241,7	108,2	14,1	34,2	mpnst
117	male	68	319,5	806,9	15,4	75,0	mpnst
118	female	50	247,1	84,3	13,8	99,1	mpnst
119	male	36	296,9	133,2	15,3	282,7	mpnst
120	female	34	108,8	228,2	16,7	26,6	mpnst
121	male	29	181,4	281,0	11,4	102,3	mpnst
122	male	49	75,5	796,6	10,0	41,7	mpnst
123	female	47	466,7	806,9	25,4	32,3	mpnst
124	female	34	270,7	143,6	13,0	140,9	mpnst
125	female	32	226,9	806,9	14,0	131,3	mpnst

Serum concentration in pg/ml

Tumor Volume
no: <10 ml
medium: 50 - 300 ml
high: > 500 ml
mpnst

Supplement Table 1: Raw data of the cohort of 125 NF1 patients.
